# Immune Factors, Immune Cells and Inflammatory Diseases

**DOI:** 10.3390/ijms25042417

**Published:** 2024-02-19

**Authors:** Alister C. Ward

**Affiliations:** 1 School of Medicine, Deakin University, Geelong, VIC 3216, Australia; alister.ward@deakin.edu.au; 2 Institute for Mental and Physical Health and Clinical Translation, Deakin University, Geelong, VIC 3216, Australia

The immune system comprises distinct innate and adaptive arms, each of which contains many layers to provide a coordinated, sequential immune response to insults [1,2]. The cells underpinning these arms of the immune system develop in multiple waves and locations throughout the life course through defined developmental pathways to yield a complex set of specialized cells across both myeloid and lymphoid lineages [3,4]. These immune cells collectively serve to maintain health throughout the life course, which includes the elicitation of transient inflammation when appropriate in response to various insults [5]. However, chronic inflammation is pathogenic and underpins a number of disease states [6,7].

The development and function of immune cells, extending to the regulation of inflammation, is moderated by a rich network of immune factors, notably including cytokines, chemokines, growth factors and other signaling proteins [8,9,10], as well as the numerous molecules that serve to regulate their action [11,12,13]. These proteins are required for normal immune cell development and function, but many are also perturbed in a variety of inflammatory disease states [14,15,16].

This Special Issue brings together a diverse set of authors and topics to consider ‘Immune Factors, Immune Cells and Inflammatory Diseases’ from a variety of perspectives. This includes the delineation of the role played by specific factors and regulators in the development and function of particular immune cell populations, such as the integrin lymphocyte function-associated antigen 1 (LFA-1) in regulatory T cells [17] and the negative feedback regulator cytokine-inducible SH2-domain containing (CISH) protein in myeloid cells [18]. This further extends to the function of such regulators in disease, specifically with respect to the cytokine vascular endothelial growth factor (VEGF) and the chronic inflammation associated with asthma [19]. The other contributions to this Special Issue explore the role of cytokine receptor-associated Janus kinase (JAK) proteins in keratinocyte susceptibility to virus infection that is relevant to various immune-related diseases, the cell surface LY108 molecule in lupus-prone mice and biomarkers of immunogenic cell death in the context of severe acute pancreatitis. 

Together, these articles have added to our understanding of this important area of study. Such research has proven to have direct clinical significance, underpinned by the exciting results already seen in modulating immune factors in relevant inflammatory and other diseases [20,21].
